# Bio-generated CoO-NPs from *Salvia officinalis*: a promising tool against ESBL-producing bacteria

**DOI:** 10.1038/s41598-026-52141-2

**Published:** 2026-05-19

**Authors:** Mohamed H. Kalaba, Ahmed A. Elrefaey, Mohamed E. Saber, Mohamed H. Sharaf, Saad A. Moghannem, Amira Salah El-Din Youssef

**Affiliations:** 1 Botany and Microbiology Department, Faculty of Science, Al-Azhar University, Cairo, Egypt; 2 Virology and immunology Unit, Cancer Biology department, National Cancer Institute, Cairo University, Giza, Egypt

**Keywords:** Extended-spectrum β-lactamase, ESBL phenotypic and biochemical screening, *Salvia officinalis*, CoO-NPs Biosynthesis, VERO and OEC cells, Nanobiotechnology, Antimicrobials, Applied microbiology, Clinical microbiology

## Abstract

Extended-spectrum β-lactamase (ESBL)-producing bacteria pose significant therapeutic challenges, necessitating the development of alternative antimicrobial agents such as metal nanoparticles. Among 58 bacterial isolates from clinical samples at the National Cancer Institute (Cairo, Egypt), eight ESBL-producing strains were identified (six *E. coli* and two *K. pneumoniae*) using phenotypic screening and VITEK 2 system, representing preliminary laboratory findings. GC-MS analysis of *Salvia officinalis* aqueous extract revealed 16 compounds, with rosmarinic acid (20.41%) as the major constituent. The successful green synthesis of cobalt oxide nanoparticles (CoO-NPs) using *S. officinalis* was confirmed through UV-visible spectroscopy showing a characteristic peak at 520 nm, HRTEM revealing particle sizes of 10–50 nm, and XRD patterns matching the CoO phase. FTIR spectroscopy confirmed the presence of metal-oxygen bonds and surface functionalization. CoO-NPs demonstrated significant antibacterial activity against ESBL-producing isolates with inhibition zones ranging from 24 to 26 mm and MIC values of 0.312–0.625 mg/ml while enhancing the efficacy of several antibiotics, particularly rifampicin, meropenem, and gentamicin. Antioxidant assays revealed free radical scavenging activity with IC50 values of 513.7 µg/mL and 208 µg/mL for DPPH (2,2-diphenyl-1-picrylhydrazyl) and ABTS (2,2′-azino-di-[3-ethylbenzthiazoline sulfonate (6)]), respectively. Cytotoxicity studies against normal cell lines showed dose-dependent effects with IC50 values of 303.526 µg/mL for VERO cells and 253.19 µg/mL for OEC cells, with significant morphological changes observed at 500 µg/mL. These preliminary laboratory findings suggest that *S. officinalis*-mediated CoO-NPs represent a promising therapeutic agent against ESBL-producing bacteria, offering a balance between antimicrobial efficacy and biological safety.

## Introduction

Antimicrobial resistance represents a major and escalating challenge in clinical practice, particularly due to the increasing prevalence of extended-spectrum β-lactamase (ESBL)-producing bacteria. Pathogens such as *Escherichia coli* and *Klebsiella pneumoniae* exhibit resistance to a broad range of β-lactam antibiotics, significantly limiting therapeutic options and complicating infection management^1^. This resistance is primarily mediated by β-lactamase enzymes, which hydrolyze the β-lactam ring of penicillins and cephalosporins. Although β-lactamase inhibitors such as clavulanate, tazobactam, and sulbactam can partially restore antibiotic activity, the continued emergence and dissemination of ESBL-producing strains in both human and animal populations over the past two decades has intensified the global resistance burden^2^.

The development of novel antibiotics is urgently needed to address this threat; however, traditional drug discovery is time-consuming, costly, and associated with high failure rates^3^. Consequently, alternative and complementary strategies to combat antibiotic resistance are being actively explored. In this context, nanotechnology has gained increasing attention in pharmaceutical and biomedical research due to its unique physicochemical properties and potential for enhanced antimicrobial efficacy.

Green nanotechnology, which employs biological resources such as plant extracts for nanoparticle synthesis, offers an eco-friendly and sustainable approach. Plant-mediated synthesis not only avoids toxic chemicals but also enables the incorporation of bioactive phytochemicals that can influence nanoparticle size, stability, and biological activity^4^. Medicinal plants are rich sources of secondary metabolites, including alkaloids, flavonoids, saponins, tannins, terpenoids, glycosides, and phenolic compounds, which can act as effective reducing and stabilizing agents during nanoparticle formation^5^.

*Salvia officinalis* L. (sage) is a well-known medicinal plant characterized by its distinct balsamic and camphoraceous aroma, attributed to its rich phytochemical profile. Sage and its derivatives have been widely used in traditional and modern medicine for the management of various conditions, including diabetes, cancer, anxiety, and cognitive disorders, and are known to possess anti-inflammatory, antioxidant, and neuroprotective properties^6^.

Cobalt oxide nanoparticles (CoO-NPs) have emerged as promising candidates for biomedical applications due to their small size, high surface area, and multifunctional properties. They have been investigated for use in drug delivery systems, antimicrobial and antifungal therapies, and wound healing applications^7–9^. The antimicrobial activity of CoO-NPs is largely attributed to their ability to generate reactive oxygen species (ROS), which can disrupt bacterial cell membranes and inhibit cellular functions. Nevertheless, concerns regarding their cytotoxicity particularly at higher concentrations necessitate careful synthesis and dose optimization. Interestingly, controlled cytotoxic effects against certain cancer cell lines suggest potential applications in cancer therapy^10^.

In the present study, fresh green *Salvia officinalis* L. leaves were utilized as natural reducing and stabilizing agents to achieve controlled biosynthesis of CoO-NPs. The antibacterial activity of the synthesized CoO-NPs, as well as their modulatory effects on the efficacy of selected antibiotics, was evaluated against ESBL-producing bacterial isolates. By exploiting the polyphenolic and terpenoid composition of *S. officinalis*, this work addresses a specific gap in green nanotechnology research, offering a targeted strategy for the bioreduction and stabilization of CoO-NPs with enhanced activity against multidrug-resistant pathogens, beyond the generalized approaches described in recent reviews^11^.

## Materials and methods

### Chemicals and reagents

All chemicals and reagents used in this study were of analytical grade. MacConkey agar and Luria–Bertani (LB) broth supplemented with 50% glycerol were obtained from Thermo Fisher Scientific (USA). Mueller–Hinton agar (MHA) and Mueller–Hinton broth (MHB), as well as antibiotic discs including amoxicillin/clavulanic acid (AMC, 20/10 µg), cefotaxime (CTX, 30 µg), ceftazidime (CAZ, 30 µg), ceftriaxone (CRO, 30 µg), and meropenem (MEM, 10 µg), were purchased from Oxoid (UK). Additional reagents included ampicillin, penicillin G, phosphate buffer solutions (0.01 M and 0.1 M), potassium iodide, iodine–iodide reagent, starch, resazurin solution (0.02% w/v), cobalt nitrate hexahydrate [Co(NO₃)₂·6 H₂O], 2,2-diphenyl-1-picrylhydrazyl (DPPH), ABTS reagent, ascorbic acid (HiMedia, Mumbai, India), RPMI medium, and MTT reagent (Bio Basic Canada Inc.).

### Isolation of Gram-negative (G-ve) bacteria

Thirty-five clinical specimens, including urine, wound exudates, stool, sputum, and blood samples, were collected from the National Cancer Institute (NCI), Cairo University, Egypt, for the isolation of pathogenic Gram-negative bacteria. Samples were cultured on nutrient agar plates and incubated at 37 °C for 24–48 h. Pure isolates were obtained by serial streaking on MacConkey agar, after which single colonies were inoculated into LB broth supplemented with 50% glycerol and stored at − 20 °C for subsequent analyses^12^.

The study protocol was approved by the Institutional Review Board of the National Cancer Institute, Cairo University, Egypt (approval no. CB2309-302-071). Written informed consent was obtained from all participants prior to sample collection. All procedures involving human participants were performed in accordance with the ethical standards of the institutional research committee and with the Declaration of Helsinki.

### Screening of ESBL-producing isolates

Initial screening for ESBL production was performed according to Mahfouz et al.^13^ using cefotaxime (CTX, 30 µg), ceftazidime (CAZ, 30 µg), and ceftriaxone (CRO, 30 µg). Bacterial isolates showing resistance to at least one of the tested cephalosporins were considered potential ESBL producers. Plates were incubated at 37 °C. Isolates were classified as resistant if inhibition zones were < 27 mm for CTX, < 22 mm for CAZ, or < 25 mm for CRO^14,15^.

### Confirmation of ESBL producers

ESBL production in the selected bacterial isolates was confirmed using phenotypic and biochemical methods, as well as the VITEK 2 system.

### Phenotypic detection

ESBL production was confirmed using the disk synergy test on Mueller–Hinton agar as described previously by Mahfouz et al.^13^. Discs of CTX, CAZ, and CRO were placed equidistantly around an amoxicillin/clavulanic acid (AMC, 20/10 µg) disc on inoculated agar plates. Following incubation at 37 °C for 18–24 h, isolates showing enhancement of inhibition zones toward the AMC disc were considered ESBL producers^16^.

### Biochemical detection using the iodometric assay

#### Extraction of crude beta-lactamase enzyme

Suspected ESBL-producing isolates were cultured in LB broth supplemented with ampicillin (20 µg/mL) at 37 °C for 18–24 h. Cells were harvested by centrifugation (10,000 rpm, 15 min, 4 °C) and washed twice with 0.01 M phosphate buffer (pH 7.0). Cell lysis was achieved by three freeze–thaw cycles, followed by centrifugation at 13,000 rpm for 20 min at 4 °C. The supernatant containing crude β-lactamase was collected and stored at − 20 °C.

#### Detection of β-Lactamase Activity using the Iodometric Assay

Activity of β-Lactamase was determined by monitoring the hydrolysis of penicillin G using an iodometric assay^17^. Reaction mixtures contained starch-iodine solution, penicillin G substrate (6 mg/mL in 0.1 M phosphate buffer, pH 6), and crude enzyme extract. Absorbance was measured at 620 nm at regular intervals up to 50 min. A color change from blue to colorless indicated positive β-lactamase activity. All assays were performed in triplicate.

#### Identification of ESBL-producing bacteria using the VITEK 2 system

Final identification of ESBL-producing isolates was performed using the VITEK 2 automated system (BioMérieux, USA) according to the manufacturer’s instructions at the Clinical Pathology Department, El-Kasr El-Ainy Hospital, Cairo, Egypt.

### Preparation of plant extract

*Salvia officinalis* L. (sage), a member of the Lamiaceae family, was collected from Saft El-Laban Plant Nursery, Giza Governorate, Egypt, and taxonomically authenticated by Prof. Dr. Abdo Marey (Faculty of Science, Al-Azhar University).

The identified samples were deposited in a publicly accessible herbarium (ID: A.P.421/2024).

The collection and experimental use of *S. officinalis L*. were carried out in full compliance with the relevant institutional, national, and international guidelines and applicable legislation. Since the plant material was sourced from a licensed commercial nursery and represents a cultivated, non-endangered species, no specific collection permit or formal ethical approval was required.

The leaves were washed with distilled water, shade-dried for three days, then (10 g in 200 mL distilled water) at 60 °C for 2 h. After cooling, the extract was filtered using Whatman No. 1 filter paper. A portion was air-dried at 50 °C for GC–MS analysis^18^, while the remaining extract was stored at 4 °C until use.

All experimental procedures and methods were performed in accordance with the relevant guidelines and regulations.

### Gas chromatography/mass spectrometry (GC/MS)

GC–MS analysis was conducted using a Trace GC-1310 ISQ system (Thermo Scientific, USA) equipped with a TG-5MS capillary column. Operating conditions followed established protocols^19^, and compound identification was achieved by comparison with WILEY 09 (Wiley, New York, NY, USA) and NIST 11 (National Institute of Standards and Technology, Gaithersburg, MD, USA) libraries.

### Biosynthesis and characterizations of cobalt oxide nanoparticles (CoO-NPs)

The CoO-NPs were synthesized using a green method with an aqueous extract of *Salvia officinalis* leaves as a reducing agent. Equal volumes (50 mL) of the leaf extract and 1 mM Co(NO₃)₂·6 H₂O solution were mixed at room temperature and stirred at 150 rpm for 3 h in the dark, with color change indicating nanoparticle formation. UV–visible spectra of the extract, cobalt salt, and reaction mixture were recorded between 200 and 800 nm (Unico 2100, USA).

To identify the functional groups involved in the reduction and stabilization of CoO-NPs, the biosynthesized nanoparticles were characterized by Fourier Transform Infrared (FTIR) spectroscopy in diffuse reflectance mode following the method described in^20^. FTIR spectra were recorded using an Agilent Cary 630 spectrometer (Faculty of Science, Al-Azhar University, Cairo, Egypt) over the range of 4000–400 cm⁻¹. The crystalline structure of the CoO-NPs was analyzed by X-ray diffraction (XRD) using a Shimadzu diffractometer equipped with Cu-Kα radiation and a nickel filter at the National Center for Radiation Research and Technology (NCRRT), Cairo, Egypt. Additionally, the morphology and selected area electron diffraction (SAED) patterns were examined by high-resolution transmission electron microscopy (HR-TEM) (JEOL 2100, Japan) at the National Research Center (NRC), Giza, Egypt. The surface charge (zeta potential) of CoO-NPs. was determined using a dynamic light scattering (DLS)-based Zetasizer Nano Series (Nano ZS, Malvern Instruments Ltd., Malvern, UK).

### Antibacterial activity and determination of minimum inhibitory concentration (MIC) of CoO-NPs

The antibacterial activity of biosynthesized CoO-NPs (1 mg/mL) was evaluated against ESBL-producing isolates and *E. coli* ATCC 25,922 using the agar well diffusion method^21^. Bacterial suspensions adjusted to 0.5 McFarland (~ 1 × 10⁶ CFU/mL) were spread on Mueller–Hinton agar plates. Wells (8 mm) were filled with 50 µL of plant extract, cobalt salt solution (1 mM Co(NO₃)₂·6 H₂O), or CoO-NPs (1 mg/mL). Meropenem (10 µg) and ceftriaxone (30 µg) served as reference antibiotics. Plates were incubated at 37 °C for 24 h, and inhibition zones were measured. Experiments were performed in triplicate.

The MIC of CoO-NPs was determined by the broth microdilution method according to (El-Didamony et al.., 2024)^22^.

### Effect of CoO-NPs on the sensitivity of bacteria against antibiotics

To assess the effect of CoO-NPs on bacterial antibiotic susceptibility, two isolates from each genus were tested with and without CoO-NPs pretreatment at a sublethal dose (0.5 MIC). Each isolate was cultured in LB broth (20 mL) in two flasks: one supplemented with CoO-NPs (0.5 MIC) and one untreated control. Cultures were incubated at 37 °C with shaking at 120 rpm for 18 h. Following incubation, bacterial suspensions were adjusted to 0.5 McFarland standard, and antibiotic susceptibility was evaluated on Mueller–Hinton agar using the disk diffusion method.

The antibiotics tested included ciprofloxacin, levofloxacin, nitrofurantoin, amikacin, rifampin, trimethoprim–sulfamethoxazole, meropenem, cefepime, ampicillin–sulbactam, gentamicin, piperacillin–tazobactam, and amoxicillin. Plates were incubated at 4 °C for 2 h, followed by 24 h at 37 °C. Inhibition zones were measured in millimeters, and results were expressed as mean values from three independent experiments.

### Antioxidant activity

The antioxidant activity of CoO-NPs was evaluated using DPPH and ABTS radical scavenging assays following the method of Shehata et al.^18^. Using the following equations.

$$\begin{gathered} DPPH\;scavenging\;activity=\frac{{control\;absorbance - CoO{\mathrm{-}}NPs\;absorbance}}{{control\;absorbance}} \times 100 \hfill \\ APTS\;scavenging\;activity=\frac{{control\;absorbance - CoO{\mathrm{-}}NPs\;absorbance}}{{control\;absorbance}} \times 100 \hfill \\ \end{gathered}$$

### Cytotoxicity of CoO-NPs against normal cell lines

The cytotoxicity of CoO-NPs was evaluated using two normal cell lines: African green monkey kidney (VERO) and oral epithelial cells (OEC), following the method described by El-Sherbiny et al.^23^. Cells, obtained from Nawah Scientific Inc., Cairo, Egypt, were seeded as 24-hour-old monolayers and treated with varying concentrations of CoO-NPs (31.25–1000 µg/mL) in 100 µL RPMI medium containing 2% serum. After 24 h incubation at 37 °C in a 5% CO₂ incubator, 20 µL/well of MTT solution (5 mg/mL in PBS; Bio Basic, Canada) was added, shaken for 5 min, and incubated for an additional 4 h. The medium was then removed, 200 µL DMSO was added to dissolve formazan crystals, and absorbance was measured at 560 nm. Cell viability (%) was calculated as:

$${\text{Cell viability }}\left( \% \right)={\text{OD of treated cells}}\backslash {\text{OD control}} \times {\mathrm{1}}00$$

IC₅₀ values were determined for each cell line. Morphological changes in the cells were also observed using an inverted phase-contrast microscope after 24 h exposure to CoO-NPs.

### Statistical analysis

All measurements were conducted in triplicate. Results are expressed as mean ± standard deviation (SD) and analyzed using Minitab 18 and Microsoft Excel 365.

## Results and discussion

### Isolation of Gram-negative bacteria and screening of the ESBL-producing isolates

A total of 58 bacterial isolates were recovered from clinical samples collected at the National Cancer Institute (NCI), Cairo, Egypt. The isolates originated from different clinical specimens, including urine (*n* = 22), wound swabs (*n* = 6), stool (*n* = 3), sputum (*n* = 2), and blood (*n* = 2). Primary isolation was performed on nutrient agar, followed by subculturing on MacConkey agar to selectively eliminate Gram-positive bacteria. Among the recovered isolates, 35 exhibited growth on MacConkey agar and were subsequently subjected to Gram staining, which confirmed that all were Gram-negative bacteria.

**Fig. 1:**
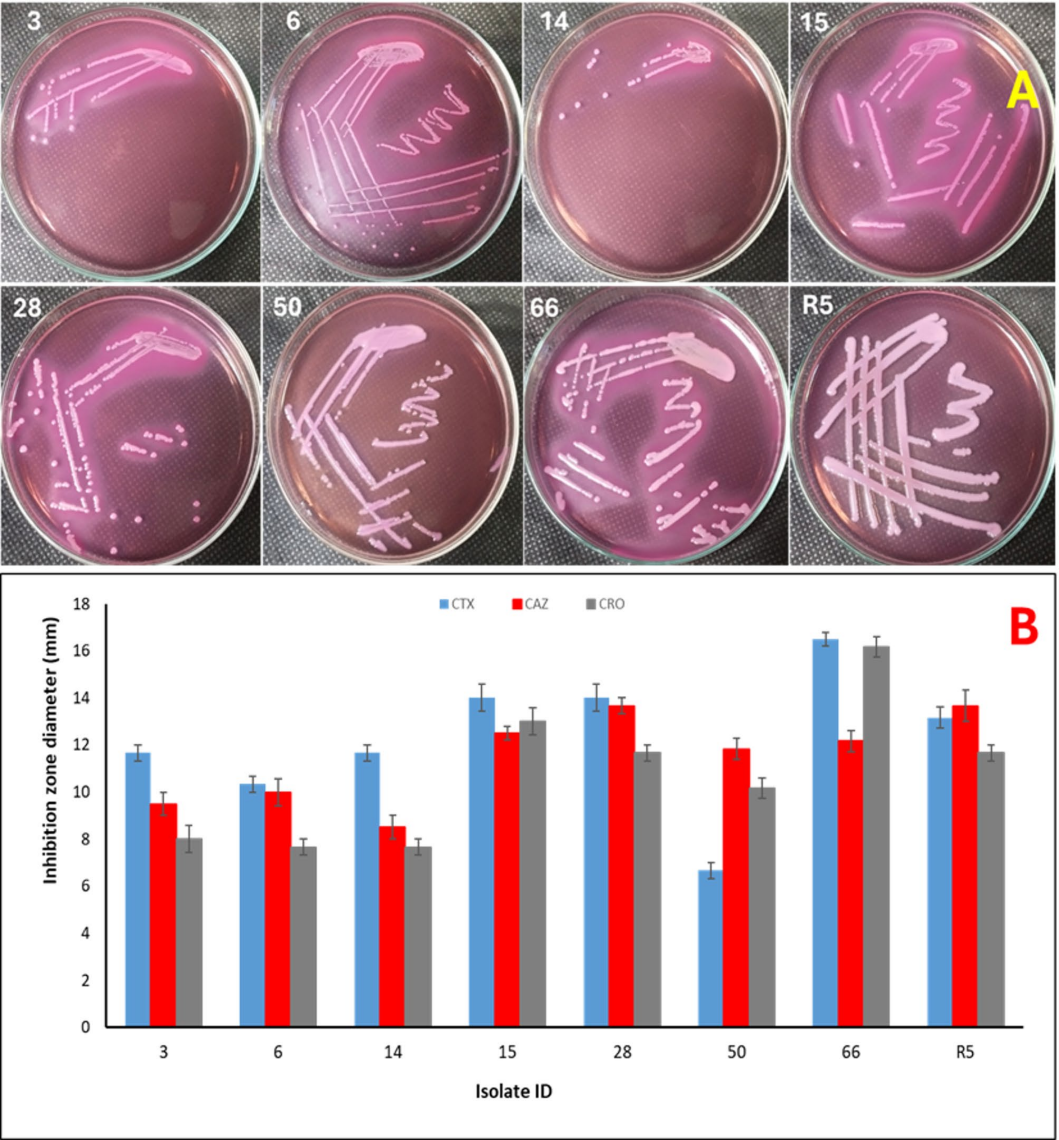
Phenotypic identification of ESBL-producing Gram-negative bacterial isolates. (**A**) Growth of ESBL-producing isolates on MacConkey agar plates. (**B**) Inhibition zone diameters used for phenotypic detection of ESBL production based on CLSI criteria.

Phenotypic screening for extended-spectrum β-lactamase (ESBL) production revealed that only eight isolates (coded as 3, 6, 14, 15, 28, 50, 66, and R5) met the Clinical and Laboratory Standards Institute (CLSI) criteria for ESBL suspicion, as evidenced by the absence of inhibition zones or zone diameters smaller than the recommended breakpoints. Figure 1A and B illustrate the growth of these isolates on MacConkey agar and the phenotypic detection of ESBL production, respectively, based on inhibition zone diameters obtained with cefotaxime (CTX), ceftazidime (CAZ), and ceftriaxone (CRO).

Isolates 3, 6, 14, and 50 demonstrated markedly reduced inhibition zones for all tested antibiotics, ranging from 7 to 11 mm, indicating a high level of resistance. In contrast, isolates 15, 28, and R5 showed moderate inhibition zone diameters ranging from 11 to 14 mm. Isolate 66 exhibited comparatively larger inhibition zones, measuring between 13 and 17 mm, suggesting a relatively lower degree of resistance.

Among the 35 Gram-negative bacterial isolates recovered from diverse clinical specimens, including urine, wound exudates, stool, sputum, and blood, only eight isolates (22.8%) were identified as extended-spectrum β-lactamase (ESBL) producers, as indicated by inhibition zone diameters below the CLSI-defined susceptibility thresholds for third-generation cephalosporins. According to CLSI guidelines^24^, the inhibition zone diameter breakpoints used for phenotypic detection of ESBL production are ≤ 27 mm for cefotaxime (CTX), ≤ 22 mm for ceftazidime (CAZ), and ≤ 25 mm for ceftriaxone (CRO).

The prevalence of ESBL-producing isolates observed in the present study is consistent with findings reported in some previous studies conducted within the region. For example, El-Masry et al.^15^ reported an ESBL prevalence of 26.8% among 138 tested clinical isolates. In contrast, other studies have documented substantially higher ESBL prevalence rates. Moreover, Shaaban et al.^25^, found that 34 out of 58 Proteus mirabilis isolates (58.6%) were β-lactamase producers. The comparatively lower prevalence of ESBL-producing isolates detected in the current study (22.8%) suggests that ESBL distribution among clinical isolates may vary according to bacterial species, specimen type, and geographic location^26^.

### Confirmation of ESBL producers

#### Phenotypic detection

**Fig. 2:**
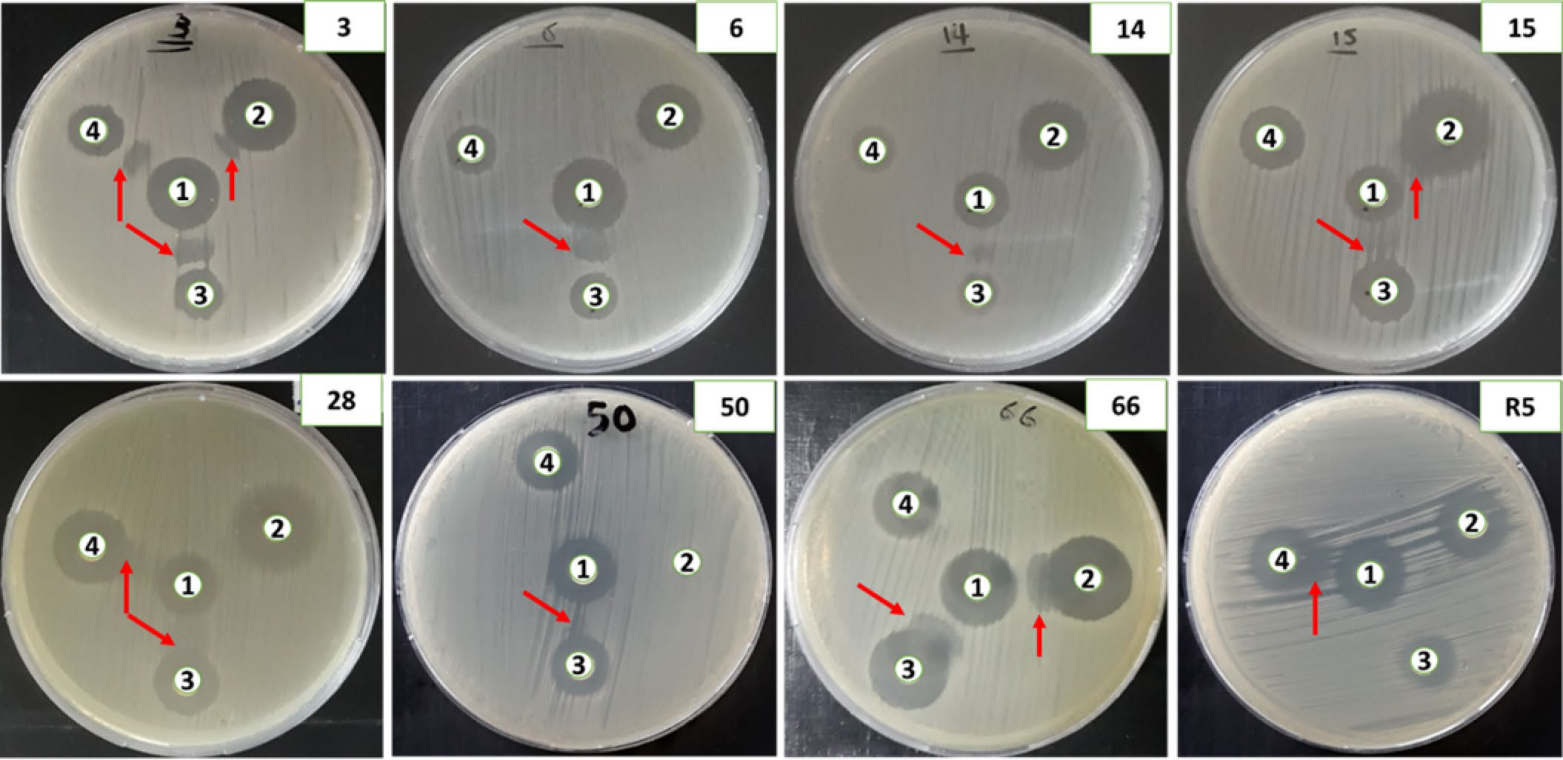
Phenotypic confirmation of ESBL production using the combined disc diffusion method. Discs used include: (1) amoxicillin/clavulanic acid (AMC), (2) cefotaxime (CTX), (3) ceftriaxone (CRO), and (4) ceftazidime (CAZ). Synergistic enhancement of inhibition zones in the presence of clavulanate indicates ESBL production.

Phenotypic detection of ESBL production was confirmed by demonstrating synergistic interactions between third-generation cephalosporins—cefotaxime (CTX), ceftazidime (CAZ), and ceftriaxone (CRO)—and the amoxicillin/clavulanic acid (AMC) disc, a method commonly referred to as the “ESBL key,” as illustrated in Table 1 and Fig. 2. This synergistic effect was evidenced by a marked increase in inhibition zone diameters in the presence of clavulanate.

The results showed that isolates 3, 15, 28, and 66 exhibited consistent synergy with AMC when combined with all tested cephalosporins, thereby confirming their classification as ESBL producers. In contrast, isolates 6, 14, 50, and R5 demonstrated selective synergy with specific cephalosporins. Nevertheless, the observed enhancement of antibacterial activity in the presence of clavulanate confirms the presence of ESBL enzymes in all tested isolates.

**Table 1:** Phenotypic confirmation of ESBL production based on synergistic interactions between the amoxicillin/clavulanic acid (AMC) disc and third-generation cephalosporins.

Isolate ID	The synergy of AMC toward cephalosporin antibiotics			ESBL interpretation
	CTX	CRO	CAZ	
3	Yes	Yes	Yes	Yes
6	No	Yes	No	Yes
14	No	Yes	No	Yes
15	Yes	Yes	No	Yes
28	No	Yes	Yes	Yes
50	No	Yes	No	Yes
66	Yes	Yes	No	Yes
R5	No	No	Yes	Yes

#### Biochemical detection using the iodometric assay

Biochemical detection of β-lactamase activity in the selected bacterial isolates was assessed using the iodometric assay. This method relies on monitoring penicillin hydrolysis over time by measuring the optical density (OD) at 620 nm, where a progressive decrease in OD reflects enzymatic degradation of penicillin and subsequent reduction in the iodine–starch complex absorbance. A sustained decline in OD values over the 50-minute assay period indicated active β-lactamase-mediated hydrolysis.

**Fig. 3:**
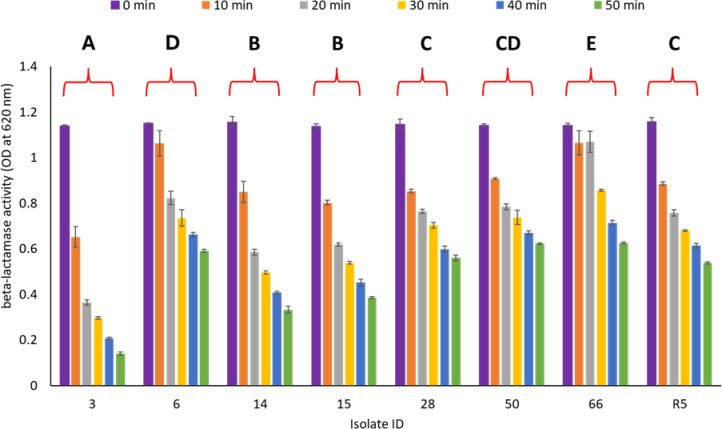
Biochemical detection of β-lactamases activity using the iodometric assay.

As shown in Fig. 3, statistical analysis using two-way repeated-measures ANOVA (RM-ANOVA) revealed significant differences in β-lactamase activity among the tested isolates (*p* < 0.01). Lowercase letters in the figure denote statistically significant differences between isolates. Isolate 3 (A) exhibited the highest β-lactamase activity, as indicated by the lowest OD values throughout the assay period. This was followed by isolates 14 and 15 (B), which showed significantly elevated β-lactamase activity compared with the remaining isolates (*p* < 0.01). In contrast, isolate 66 (E) demonstrated the lowest β-lactamase activity, with OD values significantly higher than those of all other isolates (*p* < 0.01). No statistically significant differences were observed among isolates 28, R5, 50, and 6 at most time points, suggesting comparable levels of β-lactamase production within this group.

In addition to inter-isolate variation, β-lactamase activity exhibited a significant time-dependent pattern. Mean OD values decreased progressively across all tested isolates from baseline (0 min) to 50 min, with significant reductions observed at each subsequent time point. Enzymatic activity became evident as early as 10 min and persisted throughout the assay duration, reaching maximal substrate hydrolysis at 50 min. This continuous decline in OD confirms sustained β-lactamase activity and ongoing penicillin degradation over time.

The CLSI recommends confirmatory testing for the accurate detection of extended-spectrum β-lactamase (ESBL) production, adopting a two-step diagnostic approach consisting of initial screening followed by confirmatory testing. Preliminary screening methods, such as disk diffusion assays using third-generation cephalosporins including cefotaxime (CTX), ceftriaxone (CRO), and ceftazidime (CAZ), may reveal reduced susceptibility; however, these methods alone are insufficient to conclusively establish ESBL production in the tested isolates. Therefore, confirmatory testing is essential, as recommended by CLSI guidelines^24^.

In the present study, the observed synergistic interaction between amoxicillin/clavulanic acid (AMC) and cephalosporins confirmed ESBL production in the tested isolates, a finding that was further supported by the iodometric assay. Together, these results highlight substantial variability in β-lactamase production among the bacterial isolates examined, a key factor contributing to resistance against β-lactam antibiotics. Integrating phenotypic confirmatory tests with complementary biochemical or molecular techniques for assessing β-lactamase activity enhances diagnostic accuracy by providing quantitative insights into enzyme activity^27^.

### Identification of ESBL-Producing bacteria using VITEK 2 system

ESBL-producing bacterial isolates were automatically identified using the VITEK 2 system. Six isolates (3, 6, 14, 15, 28, and 66) were identified as *E. coli*, while the other two isolates (50 and R5) were identified as *K. pneumoniae*. The probability percentage of these isolates being identified ranged from 94 to 99%, as displayed in Table 2.

ESBL-producing bacterial isolates were automatically identified using the VITEK 2 system. Six isolates (codes 3, 6, 14, 15, 28, and 66) were identified as *E. coli*, while the remaining two isolates (codes 50 and R5) were identified as *K. pneumonia.* The probability of correct identification ranged from 94% to 99%, as summarized in Table 2, indicating high confidence in species-level assignment.

**Table 2:** Identification Probabilities of ESBL-Producing bacteria.

Isolate ID	3	6	14	15	28	66	50	R5
Bacterial name	*E. coli*						*K. pneumoniae*	
Probability	94	94	95	98	99	96	96	99

The predominance of *E. coli* among ESBL producers observed in the current study is consistent with previous reports. Earlier investigations have shown that ESBL production rates were approximately 55.5% in *E. coli* and 16.5% in *K. pneumoniae*^28^. More recent large-scale surveillance data further revealed that 42.5% (4,706/11,065) of E. coli isolates and 30.2% (1,697/5,617) of *K. pneumoniae* isolates were classified as ESBL producers^29^, underscoring the widespread dissemination of ESBL determinants in these clinically important pathogens. The high identification accuracy achieved in this study (94–99%) highlights the reliability and diagnostic value of the VITEK 2 system for routine clinical microbiology applications^30^.

### Phytochemical analysis of Salvia officinalis leaf extracts by GC-MS

**Fig. 4:**
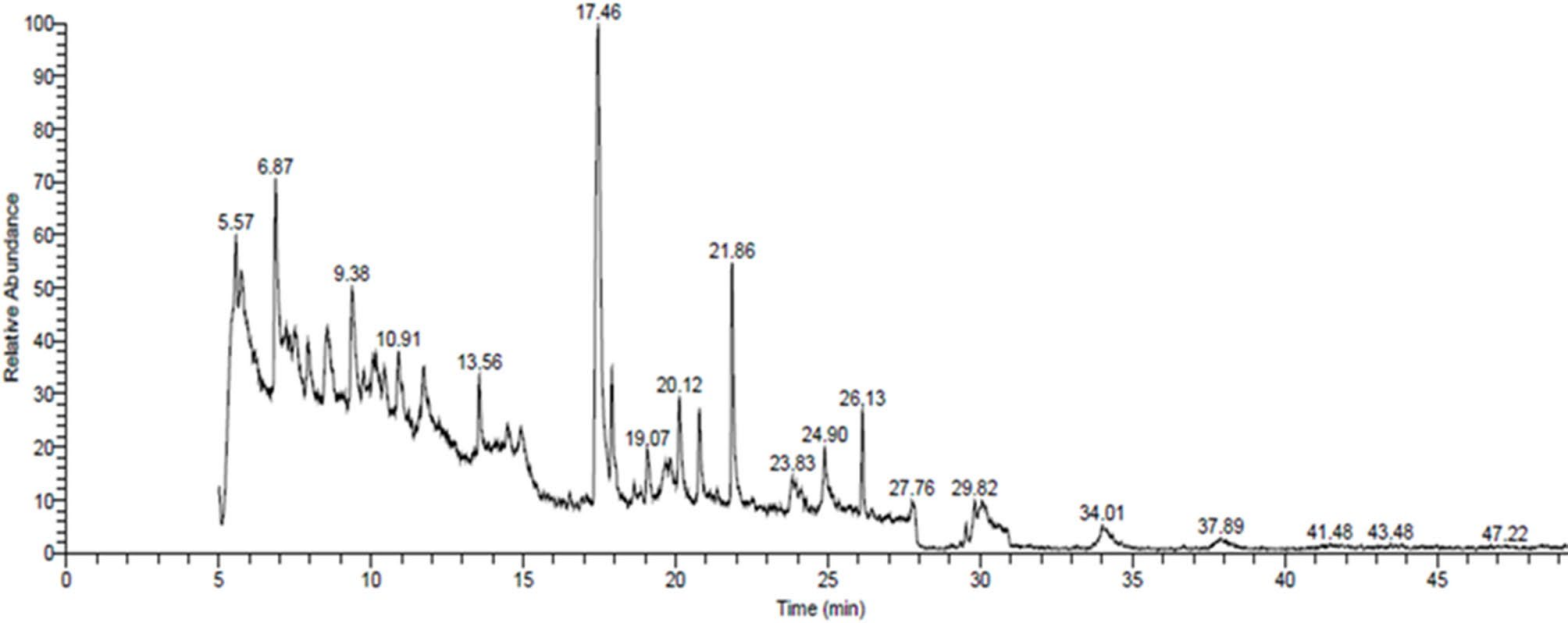
GC mass of *Salvia officinalis* L. crude extract.

Gas chromatography–mass spectrometry (GC–MS) analysis of the crude *Salvia officinalis* leaf extract (Fig. 4; full dataset presented in Table 3) identified sixteen phytochemical compounds with retention times ranging from 5.41 to 26.13 min. The relative abundance of the detected compounds, expressed as peak area percentages, ranged from 20.41% (highest) to 2.02% (lowest).

The identified compounds, ranked in descending order of peak area percentage, were rosmarinic acid (20.41%), 2-methylpropan-2-amine (6.24%), dihydroterpineol (5.92%), benzenemethanol, α-(1-aminoethyl)-, (R, R)- (4.62%), methyl 2-cyclopropyl-2-methylspiro[2.2]pentane-1-carboxylate (4.24%), 2,6-di-tert-butylphenol (3.85%), 10,12-octadecadiynoic acid (3.44%), (E)-4-hexadecen-6-yne (3.32%), methyl octadecanoate (2.88%), 1,2,3,3a,6,7,8,8a-octahydro-6,6-dimethylcyclopentacyclopropacyclohepten-3-one (2.99%), methyl (3-oxohexahydro-2-pentylcyclopentyl)acetate (2.79%), panaxydol (2.50%), longipinene epoxide (2.38%), 5α-androstan-16-one, cyclic ethylene mercaptol (2.33%), methyl 2-(trimethylsilyloxy)hexadec-6-enoate (2.08%), and spiro[2.9]dodeca-4,8-diene (2.02%).

The identity of each compound was confirmed by comparison of its mass spectrum with reference libraries, achieving similarity indices exceeding 85%. GC–MS is widely recognized as a robust and reliable analytical technique for profiling secondary metabolites in plant and non-plant matrices, providing detailed qualitative and semi-quantitative insights into complex chemical compositions^31^.

Rosmarinic acid was the predominant constituent of the extract, highlighting its phenolic richness. In addition, the presence of fatty acid derivatives, esters, terpenoids, amines, phenols, and steroidal compounds indicates a diverse mixture of polar and non-polar constituents. This phytochemical diversity is consistent with previous reports (Table 5) describing the antimicrobial, antioxidant, anticancer, and neuroprotective properties of *S. officinalis*, and supports the biological efficacy observed in the present study.

**Table 3:** Chemical composition of *Salvia officinalis* L. aqueous extract according to GC-MS analysis.

No.	Compound name	RT (min)	Peak area %	Molecular formula	Molecular weight (g/mol)	Activity	Ref.
1	Benzenemethanol, α-(1-aminoethyl)-, (R, R)	5.41	4.62	C_9_H_13_NO	151	Cardiac stimulant, and antiasthma agents	^32^
2	10,12-Octadecadiynoic acid	5.57	3.44	C_18_H_28_O_2_	276	Antimicrobial	^33^
3	Terpineol, Dihydro	6.87	5.92	C_10_H_20_O	156	Antimicrobial, Antioxidant, Anticancer and Antiulcer activity and Cardiovascular agent	^34^
4	Spiro[2.9]Dodeca-4,8-Diene	7.92	2.02	C_12_H_18_	162	Not reported	–
5	4-Hexadecan-6-YNE, (E)-	8.57	3.32	C_16_H_28_	220	Antimicrobial	^35^
6	2-Cyclopropyl-2-Methylspiro[2.2]Pentane-1-Carboxylic acid, Methyl Ester	9.38	4.24	C_10_H_14_O_2_	166	Not reported	–
7	2-Trimethylsiloxy-6-hexadecenoic acid, methyl ester	10.45	2.08	C_20_H_40_O_3_Si	356	Antifungal	^36^
8	Longipinen epoxide	10.91	2.50	C_15_H_24_O	220	Antimicrobial anticancer, anti-inflammatory	^37^
9	5à-Androstan-16-one, cyclic ethylene mercaptole	13.56	2.33	C_21_H_34_S_2_	350	Antimicrobial	^38^
10	Rosmarinic Acid	17.46	20.41	C_18_H_16_O_8_	360	Antioxidant, Antibacterial, Anti-inflammatory and anticancer activity	^39^
11	Phenol, Bis(1,1-Dimethylethyl)	17.90	3.85	C_14_H_22_O	206	Antifungal	^40^
12	Panaxydol	19.07	2.38	C_17_H_24_O_2_	260	antifatigue, antitumor activity, and neurodegenerative protection activity	^41^
13	Cyclopenta[1,3]CyclopropA[1,2]Cyclohepten-3(3AH)-ON E, 1,2,3B,6,7,8-Hexahydro-6,6-Dimethyl	20.12	2.99	C_13_H_18_O	190	Not reported	–
14	Methyl (3-OXO-2-Pentylcyclopentyl )Acetate	20.78	2.79	C_13_H_22_O_3_	226	Antimicrobial	^42^
15	2-Methyl-2-Propanamine	21.86	6.24	C_4_H_11_N	73	Not reported	–
16	Octadecanoic Acid, Methyl Ester	26.13	2.88	C_19_H_38_O_2_	298	Antiviral adjuvant	^43^

#### Biosynthesis and characterizations of CoO-NPs

**Fig. 5:**
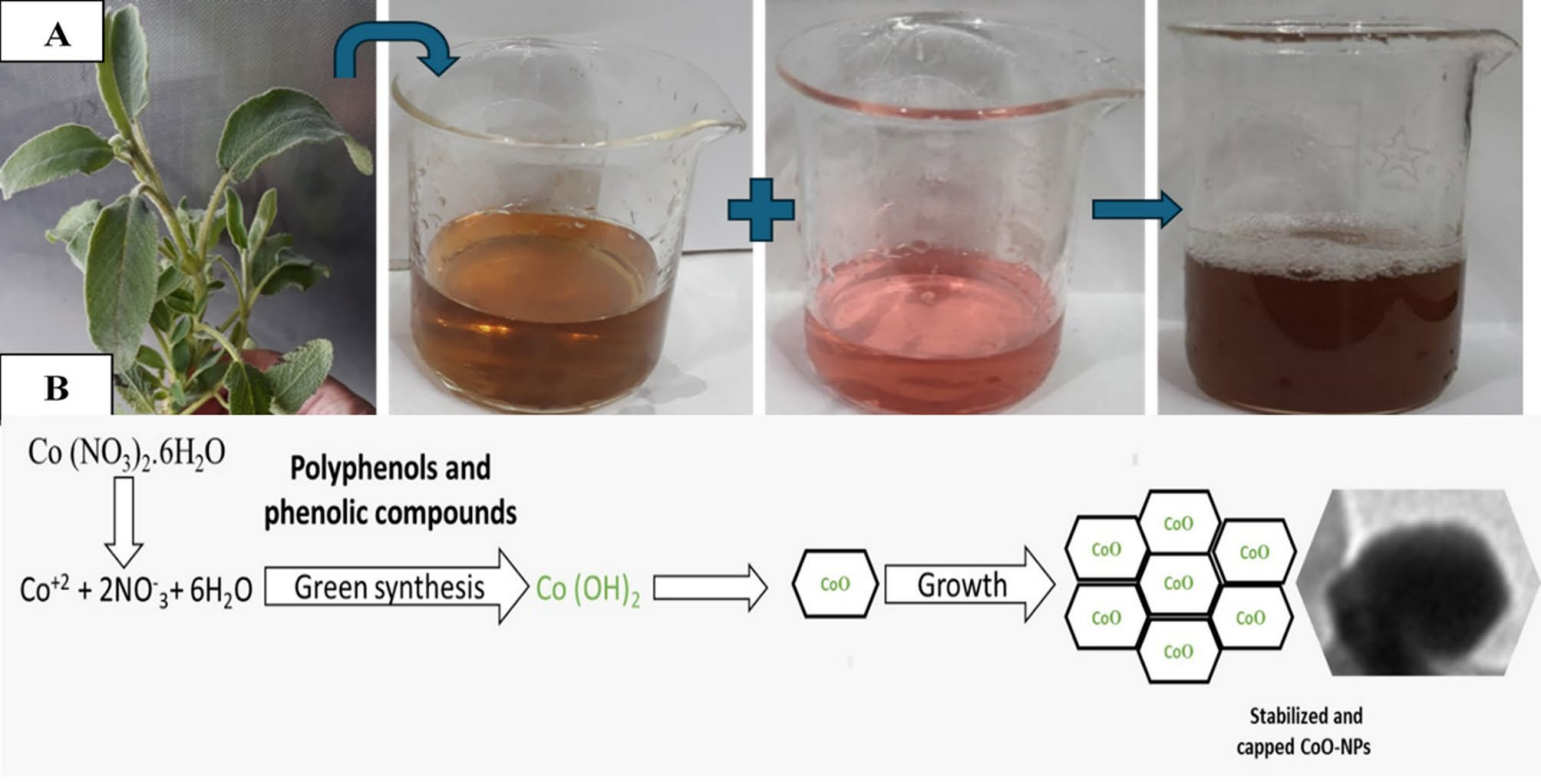
Green synthesis of CoO-NPs using *Salvia officinalis* aqueous leaf extract. (**A**) Visual representation of the biosynthesis process. (**B**) Schematic diagram illustrating the proposed mechanism of CoO-NPs formation mediated by phytochemicals in the extract.

The observed color transformation during synthesis, concluding in a homogeneous brown colloidal solution without visible precipitation, indicates successful CoO-NPs formation with adequate dispersion stability Figure 5. The plant extract demonstrates dual functionality in this synthesis pathway, simultaneously acting as a reducing medium for cobalt ion conversion and a stabilizing agent that prevents particle agglomeration^44^. The formation of CoO-NPs occurs through the reduction of cobalt (II) ions (Co²⁺) to their zero-valent state (Co⁰), a process facilitated by the bioactive compounds present in the plant extract matrix^45^.

**Fig. 6:**
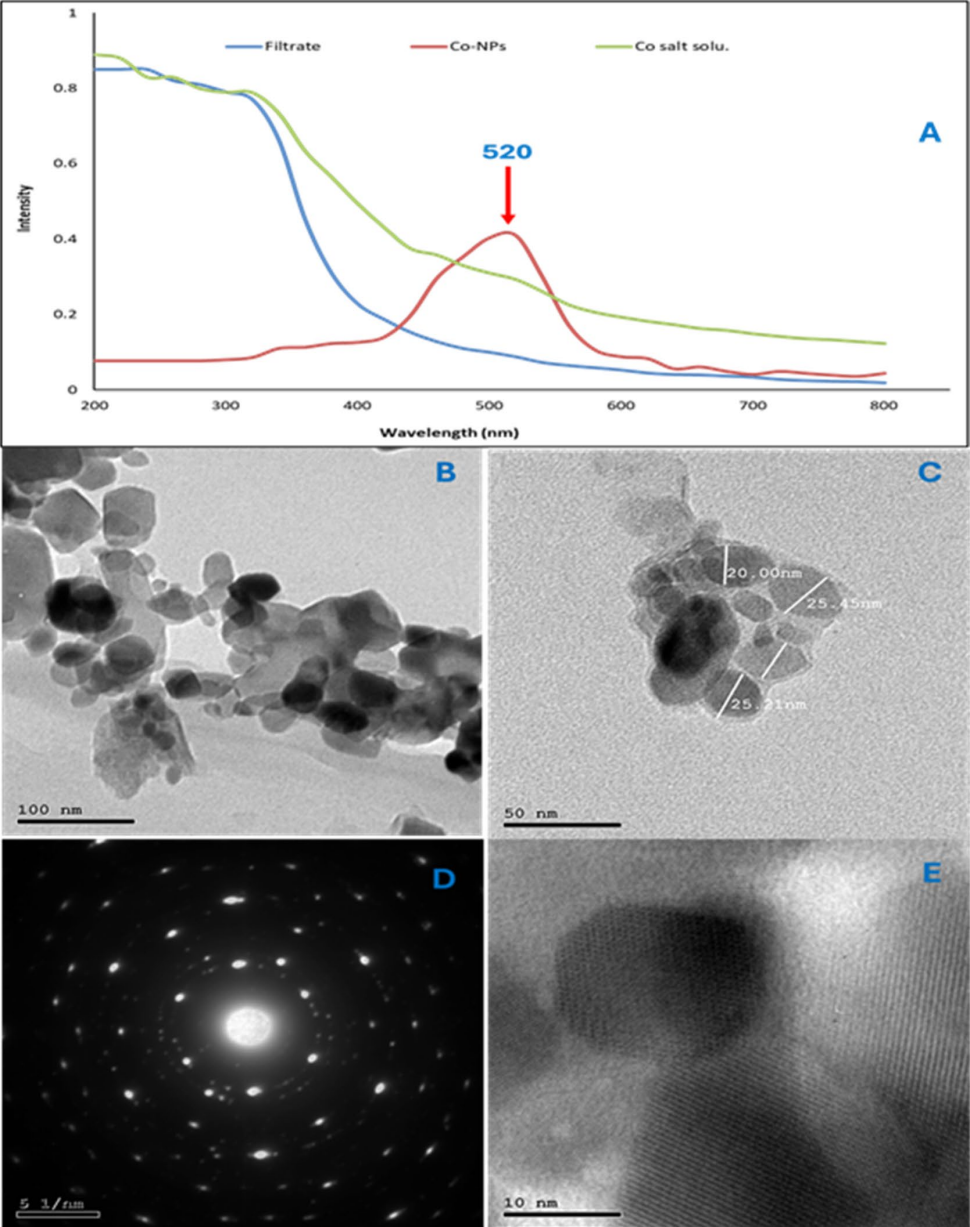
Characterization of green-synthesized CoO-NPs. (**A**) UV–visible absorption spectrum showing surface plasmon resonance at 520 nm. (**B**) High-resolution transmission electron microscopy (HR-TEM) image of CoO-NPs. (**C**) Particle size distribution of the nanoparticles. (**D**) Selected area electron diffraction (SAED) pattern indicating polycrystalline structure. (**E**) Well-defined lattice fringes observed in HR-TEM, confirming crystallinity.

The UV–visible spectrum of the synthesized CoO-NPs displayed a distinct absorption peak at 520 nm, characteristic of surface plasmon resonance (SPR), confirming the formation of CoO-NPs (Fig. 6A). In contrast, the UV–visible spectra of the filtrates from the *Salvia officinalis* aqueous extract and the cobalt precursor solution lacked any distinct absorption peaks, indicating the absence of plasmonic nanoparticles or strongly light-absorbing components. This observation confirms the effective reduction of cobalt ions and successful incorporation into CoO-NPs, with minimal residual precursor or unreacted organic material. These findings are consistent with previous reports, where green- or chemically-synthesized CoO-NPs exhibited absorption bands in the 500–530 nm range^46–48^, while variations in UV–visible peaks (220–450 nm) have been attributed to differences in synthesis methods, particle size, surface chemistry, and stabilizing agents^49–53^. The red-shifted absorption in green-synthesized nanoparticles suggests that biological reducing and capping agents influence electronic transitions and surface properties of CoO-NPs, distinguishing them from chemically synthesized counterparts.

High-resolution transmission electron microscopy (HR-TEM) revealed that the CoO-NPs possessed irregular but faceted morphologies with sizes ranging from 10 to 50 nm (Fig. 6B–E). While some particle agglomeration was observed, individual nanoparticles maintained distinct shapes. Such aggregation is common in plant-mediated nanoparticle synthesis^54^, as phytochemicals (phenolics, polysaccharides, flavonoids) simultaneously act as reducing and capping agents. These molecules facilitate surface functionalization but provide limited steric hindrance, resulting in partial inter-particle bridging. Variations in contrast within the TEM images likely reflect differences in particle thickness or minor compositional heterogeneity. Selected area electron diffraction (SAED) patterns exhibited sharp diffraction spots and concentric rings, indicating a polycrystalline structure with randomly oriented grains. Well-defined lattice fringes further confirmed the crystalline nature of the nanoparticles.

**Fig. 7:**
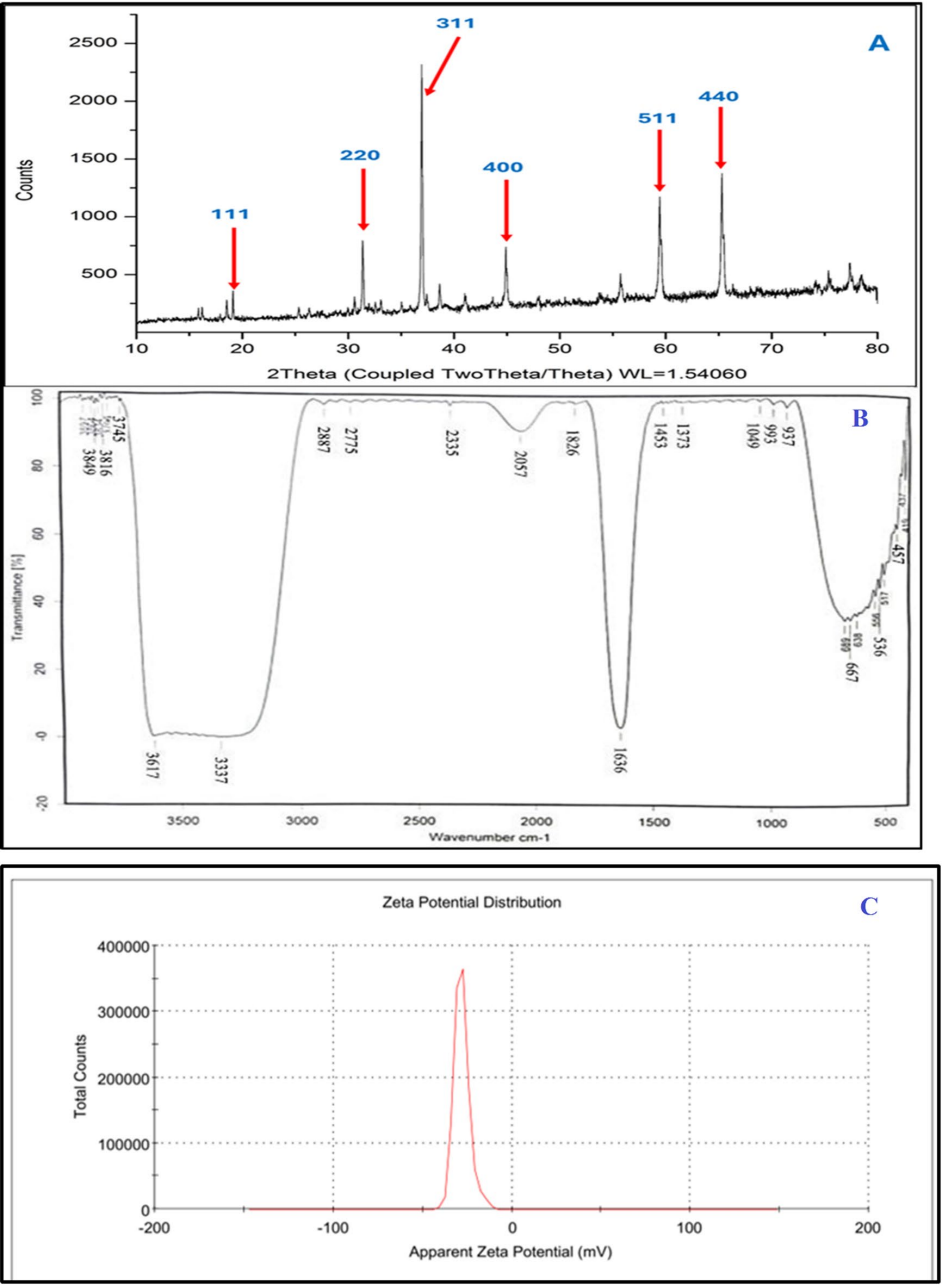
Structural and chemical characterization of green-synthesized CoO-NPs. (**A**) X-ray diffraction (XRD) pattern confirming the crystalline phase. (**B**) Fourier-transform infrared (FTIR) spectrum showing surface functional groups, including O–H, C–H, C–O, and Co–O vibrations. (**C**) Zeta potential distribution of CoO-NPs.

The average particle size, calculated using the Debye–Scherrer equation, was 22.3 ± 10.6 nm. X-ray diffraction (XRD) analysis supported these findings, revealing diffraction peaks at 2θ values of ~ 18.9°, 31.3°, 36.9°, 44.9°, 59.4°, and 65.3°, corresponding to the (111), (220), (311), (400), (511), and (440) planes of Co₃O₄ (JCPDS No. 42-1467), consistent with a cubic crystal structure (Fig. 7A)^56^. The alignment of lattice fringes in HR-TEM, the SAED patterns, and the XRD peaks collectively confirm the nanocrystalline nature, structural integrity, and phase purity of the CoO-NPs.

Fourier-transform infrared (FTIR) spectroscopy provided insights into surface functional groups (Fig. 7B). A broad band at 3437 cm⁻¹ corresponds to O–H stretching vibrations, indicating hydroxyl groups from adsorbed water or surface hydroxyls. Peaks at 2923 and 2852 cm⁻¹ are attributed to C–H stretching from residual organic compounds. The band at 2355 cm⁻¹ reflects atmospheric CO₂, while the peak near 2077 cm⁻¹ may indicate C ≡ C stretching in alkynes or minor metal–carbonyl species. A band at 1635 cm⁻¹ suggests H–O–H bending vibrations from adsorbed moisture. Peaks around 1453 and 1375 cm⁻¹ correspond to C–H bending or carbonate species (CO₃²⁻) formed via reaction with atmospheric CO₂. The 1045 cm⁻¹ peak is assigned to C–O stretching from residual carboxyl groups of capping phytochemicals. Finally, bands at 877, 669, and 579 cm⁻¹ are associated with metal–oxygen (M–O) vibrations, with the 579 and 669 cm⁻¹ peaks characteristic of Co–O bonds, confirming the formation of cobalt oxide. The value of zeta potential was − 28 mV, indicating a moderately stable colloidal system. It is well established that nanoparticle dispersions with zeta potential values between ± 20 and ± 30 mV exhibit moderate stability (Fig. 7C).

#### Antibacterial activity and minimum inhibitory concentration (MIC) of CoO-NPs

The antibacterial activities of *Salvia officinalis* leaf extract, cobalt salt solution, green-synthesized CoO-NPs, and standard antibiotics (meropenem and ceftriaxone) against ESBL-producing *E. coli* and *K. pneumoniae*, as well as non-ESBL-producing *E. coli* ATCC 25,922, are summarized in Table 4.

The *S. officinalis* extract exhibited no detectable antibacterial activity against any of the tested strains. The cobalt salt solution showed moderate activity, with inhibition zones ranging from 14.0 to 18.0 mm for ESBL-producing strains and 16 mm for the non-ESBL-producing standard strain. In contrast, CoO-NPs demonstrated significantly enhanced antibacterial activity compared to the cobalt salt, with inhibition zones of 24–26 mm for ESBL-producing strains and 25 mm for *E. coli* ATCC 25,922.

Meropenem, used as a positive control, displayed the highest activity, producing inhibition zones of 30–35 mm for ESBL-producing strains and 32 mm for the non-ESBL-producing standard strain. Ceftriaxone exhibited markedly reduced activity against ESBL-producing isolates (9–15 mm) compared to *E. coli* ATCC 25,922 (28 mm), reflecting the resistance associated with ESBL production.

**Table 4:** The antibacterial activity of the tested materials against ESBL-producing bacteria.

Organism ID	Diameter of inhibition zone (mm) ± SE				
	Filtrate	Cobalt Salt solution	CoO-NPs	MEM +ve control	CRO -ve control
*E. coli* (3)	0	15.33 ± 0.33	26.16 ± 0.60	34 ± 0.57	9 ± 0.57
*E. coli* (6)	0	16.66 ± 0.88	26.5 ± 0.76	33.33 ± 0.66	10 ± 0.57
*E. coli* (14)	0	15.16 ± 0.60	25 ± 0.57	30 ± 0.57	13.66 ± 0.66
*E. coli* (15)	0	16.33 ± 0.33	24.33 ± 0.88	34.16 ± 0.60	11.33 ± 0.88
*E. coli* (28)	0	16.33 ± 0.88	26 ± 1.0	34.16 ± 0.72	11.66 ± 0.33
*K. pneumonia* (50)	0	18 ± 0.57	26 ± 0.577	31.66 ± 0.88	10.66 ± 0.88
*E. coli* (66)	0	14 ± 0.57	26.33 ± 0.88	33.66 ± 0.66	14.33 ± 0.33
*K. pneumonia* (R5)	0	17 ± 0.57	26.66 ± 0.66	35.16 ± 0.60	15.66 ± 0.66
*E. coli* ATCC 25,922	0	16.66 ± 0.67	25.83 ± 0.44	32.66 ± 0.88	28.66 ± 0.88

The MIC values of CoO-NPs against ESBL-producing isolates, determined using the resazurin-based assay, are summarized in Table 5. The results indicate that CoO-NPs exhibit variable antibacterial efficacy across the tested strains. Specifically, *E. coli* isolates 3, 6, 14, and 66 showed MICs of 0.312 mg/mL, whereas *E. coli* isolates 15 and 28, as well as *K. pneumoniae* isolates 50 and R5, exhibited higher MICs of 0.625 mg/ml.

These findings highlight the ineffectiveness of the aqueous *Salvia officinalis* extract and the relatively moderate antibacterial activity of cobalt salt solution across different bacterial types. In contrast, CoO-NPs demonstrated enhanced antibacterial activity with a broad-spectrum effect, showing comparable efficacy against both ESBL-producing and non-ESBL-producing strains. The improved performance of CoO-NPs is likely attributable to their increased surface area, which facilitates interactions with bacterial cells and promotes the generation of reactive oxygen species (ROS), ultimately disrupting cell membranes^11,57,58^.

Meropenem exhibited the highest efficacy against both ESBL and non-ESBL strains, whereas ceftriaxone showed markedly reduced activity against ESBL-producing isolates, reflecting resistance patterns and the need for alternative therapies. Notably, CoO-NPs provided a consistent, moderate antibacterial effect across all tested strains, supporting their potential as alternative antimicrobial agents. Previous studies have reported similar antibacterial properties of CoO-NPs, including inhibition zones of 15–22 mm against *E. coli*,* Proteus spp.*, and *Staphylococcus aureus* at 100 µg/mL^59^, and Our plant-extract synthesized CoO-NPs showed MICs of 0.312–0.625 mg/mL against ESBL-producing *E. coli* and *K. pneumoniae* (Table 5), comparable to values reported for similar green-synthesized CoO-NPs (8–16 mg/mL using roots extract of *Ziziphus Oxyphylla Edgew.* Or 0.5 mg/mL using *Capparis spinosa* Fruit Extract^60,61^.

MIC values as low as 4 µg/mL for *E. coli* when synthesized using *Periconia prolifica*^62^, highlighting that efficacy can vary depending on nanoparticle synthesis conditions.

Although the MIC range observed in this study (0.312–0.625 mg/mL) is higher than that of conventional antibiotics, it is important to note that nanoparticles often exhibit distinct pharmacodynamics and mechanisms of action. The higher MIC values may reflect slower release kinetics, alternative modes of bacterial interaction, or the need for higher concentrations to overcome biofilm formation or multidrug resistance. Overall, these results underscore the potential of CoO-NPs as promising antibacterial agents, while emphasizing that their activity can be influenced by bacterial strain and synthesis methodology.

**Table 5:** Minimum inhibitory concentrations (MICs) of cobalt oxide nanoparticles (CoO-NPs) against ESBL-producing *E. coli* and *K. pneumoniae* isolates.

No	Organism	MIC of Co-NPs (µg/ml)	SI (µg/ml)
1	*Escherichia coli* (3)	312	0.8
2	*Escherichia coli* (6)	312	0.8
3	*Escherichia coli* (14)	312	0.8
4	*Escherichia coli* (15)	625	1.6
5	*Escherichia coli* (28)	625	1.6
6	*Klebsiella Pneumonia* (50)	625	1.6
7	*Escherichia coli* (66)	312	0.8
8	*Klebsiella Pneumonia* (R5)	625	1.6

#### Effect of CoO-NPs on antibiotic sensitivity in bacteria

**Fig. 8:**
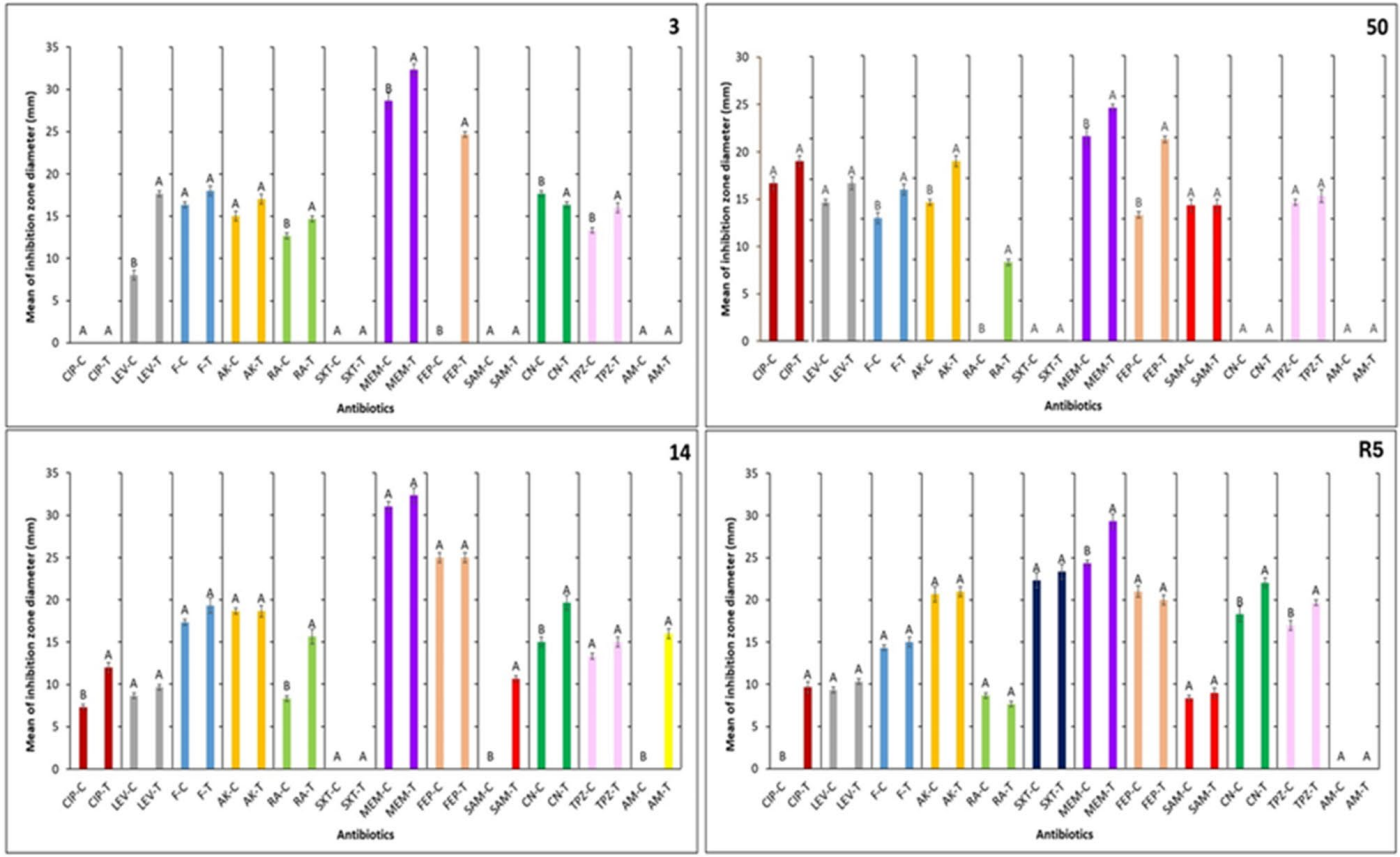
Effect of different antibiotics against pretreated ESBL-producing bacteria with a sublethal dose of CoO-NPs.

The antibiotic susceptibility profiles of two isolates from each genus (*E. coli-3* and *E. coli-14*) and (*K. pneumoniae-50* and *K. pneumoniae-R5*) were evaluated before and after exposure to sub-lethal concentrations of CoO-NPs. The results, summarized in Fig. 8, demonstrate that CoO-NPs treatment differentially influenced antibiotic sensitivity depending on the bacterial isolate and antibiotic class.

Several antibiotics showed enhanced antibacterial efficacy following CoO-NPs exposure, whereas others remained largely unaffected. Trimethoprim–sulfamethoxazole (SXT) did not exhibit any noticeable change in activity across all tested isolates, irrespective of nanoparticle treatment. In contrast, levofloxacin (LEV), nitrofurantoin (F), amikacin (AK), ampicillin/sulbactam (SAM), and amoxicillin (AM) displayed increased activity against a single isolate. Ciprofloxacin (CIP), cefepime (FEP), and piperacillin/tazobactam (TPZ) showed moderate enhancements in activity against two isolates (one from each genus).

Notably, rifampicin (RA), meropenem (MEM), and gentamicin (CN) exhibited the most pronounced improvements in antibacterial efficacy, with enhanced activity observed across multiple isolates following CoO-NPs treatment. These findings suggest that sub-lethal exposure to CoO-NPs can modulate bacterial antibiotic susceptibility in an antibiotic- and strain-dependent manner, potentially enhancing the effectiveness of selected antimicrobial agents.

SXT showed no enhancement in antibacterial activity following treatment with CoO-NPs in any of the tested isolates, likely due to intrinsic resistance mechanisms or the inability of CoO-NPs to affect the metabolic pathways targeted by this antibiotic. In contrast, increased activity observed for LEV, F, AK, SAM, and AM in individual isolates suggests isolate-specific factors influencing susceptibility.

The enhanced efficacy of LEV against *E. coli* isolate 3 may be attributed to CoO-NPs induced increases in membrane permeability, facilitating improved access to intracellular targets such as DNA gyrase and topoisomerase IV^63^. Similarly, the improved activity of F against *K. pneumoniae* isolate 50 may result from enhanced cellular uptake mediated by nanoparticle–cell interactions. The observed increases in AK, SAM, and AM activity may reflect nanoparticle-induced inhibition of resistance mechanisms or disruption of bacterial cell envelope integrity, with variability among isolates corresponding to distinct resistance profiles^64^.

Enhanced activity of CIP may be explained by improved antibiotic penetration and compromised bacterial defense systems, while the increased efficacy of FEP and TPZ suggests interference with β-lactamase-mediated resistance, particularly in ESBL-producing strains. Notably, the enhanced activity of β-lactam antibiotics such as MEM, FEP, and TPZ in selected isolates indicates that CoO-NPs may inhibit β-lactamase enzymes and compromise bacterial cell wall integrity^65^.

Collectively, Synergy was assessed qualitatively via disk diffusion zone changes post CoO-NPs pretreatment these findings highlight the potential of CoO-NPs as antibiotic adjuvants capable of enhancing the efficacy of selected antimicrobial agents against multidrug-resistant bacterial pathogens. However, the small sample size (two isolates per genus) restricts broader generalizability; larger panels of clinical strains are needed to confirm strain-independent effects and clinical relevance.

### Antioxidant activity of the green synthesized CoO-NPs

The antioxidant activity of green-synthesized CoO-NPs was evaluated using DPPH and ABTS radical scavenging assays. The results confirm that CoO-NPs exhibit notable antioxidant properties, primarily attributed to surface-bound functional groups and residual phytochemicals originating from the *Salvia officinalis* extract. These surface functionalities enhance free-radical scavenging through hydrogen atom donation and electron transfer mechanisms.

FTIR analysis supports this antioxidant behavior by revealing functional groups involved in radical neutralization. The broad absorption band around 3437 cm⁻¹ corresponds to surface hydroxyl (–OH) groups, which play a key role in hydrogen donation to quench free radicals. Peaks at 2923, 2852, 1453, and 1375 cm⁻¹ are assigned to C–H stretching and bending vibrations of organic moieties that may function as stabilizing agents with inherent antioxidant potential. The C–O stretching vibration observed at 1045 cm⁻¹ indicates the presence of carboxyl groups capable of contributing electrons for radical scavenging. Additionally, Co–O vibration bands at 579 and 669 cm⁻¹ confirm metal–oxygen bonding, which facilitates redox cycling and electron transfer processes essential for antioxidant activity.

**Fig. 9:**
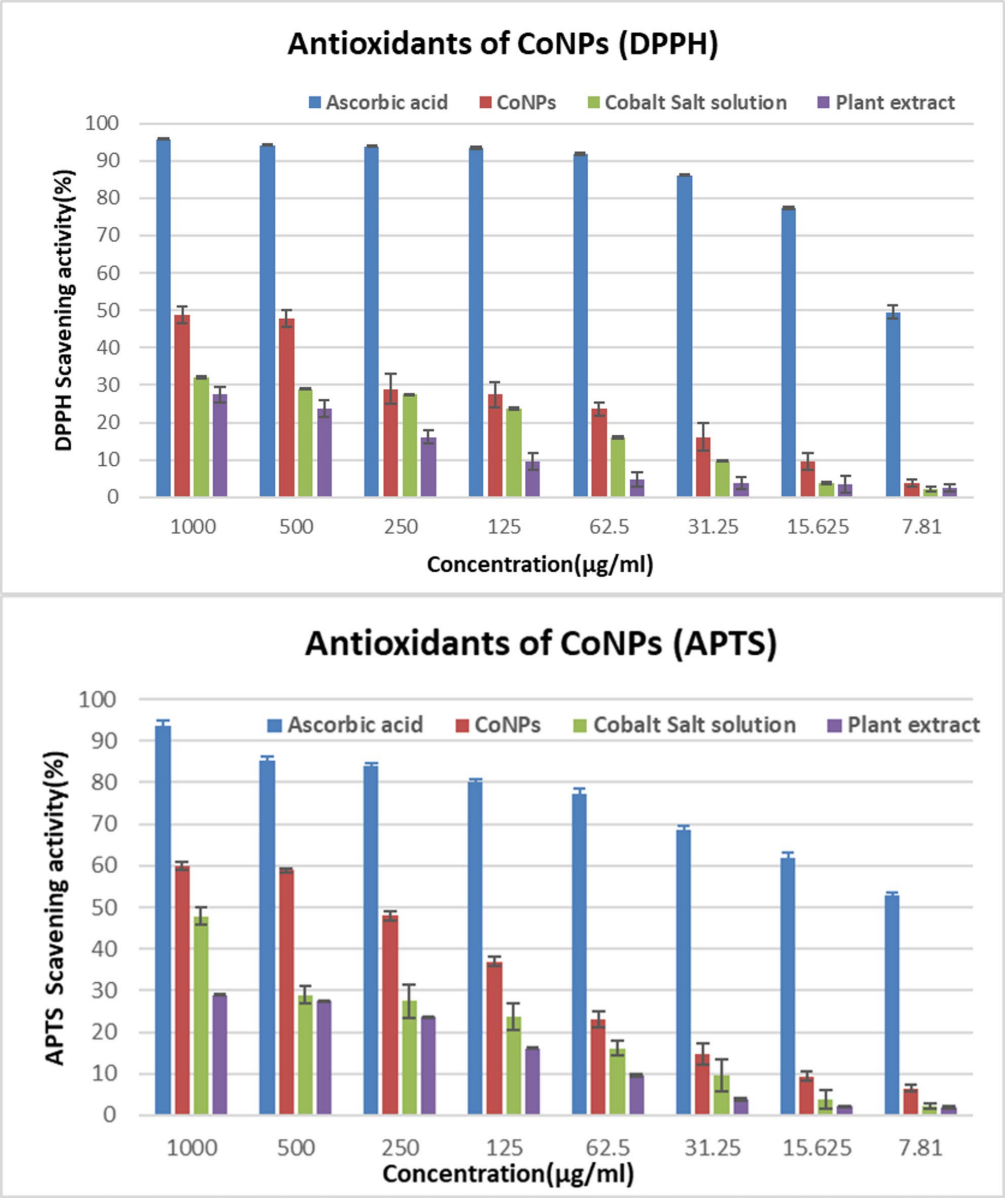
Antioxidant activity of green-synthesized CoO-NPs, filtrate, cobalt salt solution, and ascorbic acid evaluated using DPPH and ABTS assays.

At all tested concentrations, CoO-NPs demonstrated a clear concentration-dependent antioxidant response, with scavenging activity increasing as nanoparticle concentration increased. As illustrated in Figure 9, CoO-NPs exhibited moderate DPPH radical scavenging activity, achieving an inhibition of 48.46 ± 2.24% at 1000 µg/mL (IC₅₀ = 513.7 µg/mL). In comparison, the positive control, ascorbic acid, showed significantly higher activity with 95.79 ± 0.231% inhibition at the same concentration and an IC₅₀ of 9.9 µg/mL.

Similarly, in the ABTS assay, CoO-NPs reduced ABTS radicals by 59.01 ± 1.32% at 1000 µg/mL, with an IC₅₀ value of 208 µg/mL, whereas ascorbic acid achieved 93.55 ± 1.34% inhibition (IC₅₀ = 11.28 µg/mL). Notably, ABTS scavenging values were slightly higher than those obtained from the DPPH assay, indicating greater sensitivity of the ABTS method toward both hydrophilic and lipophilic antioxidants.

Previous studies have reported higher antioxidant activities for CoO-NPs synthesized using different plant extracts, underscoring the influence of synthesis conditions. For example, Co₃O₄-NPs synthesized using *Citrus tangerina* leaf extract showed 59.84% DPPH scavenging activity at 125 µg/mL^66^. Likewise, CoO-NPs synthesized with *Madhuca indica* flower extract exhibited superior antioxidant performance, attributed to the high flavonoid and phytochemical content of the extract^67^. These findings indicate that nanoparticle size, morphology, surface chemistry, and the nature of phytochemical capping agents play critical roles in determining antioxidant efficacy^68^.

Overall, both DPPH and ABTS assays consistently demonstrated that nanoparticle formation significantly enhanced antioxidant potential compared to the plant extract alone. The consistent patterns observed in both assays support the reliability of the results and show that ascorbic acid exhibited the highest antioxidant activity, followed by CoO-NPs and then the plant extract as illustrated in Fig. 9. This confirms the reliability of the assays and highlights the moderate yet promising antioxidant capacity of green-synthesized CoO-NPs.

### Cytotoxicity of CoO-NPs on normal cell lines

**Fig. 10:**
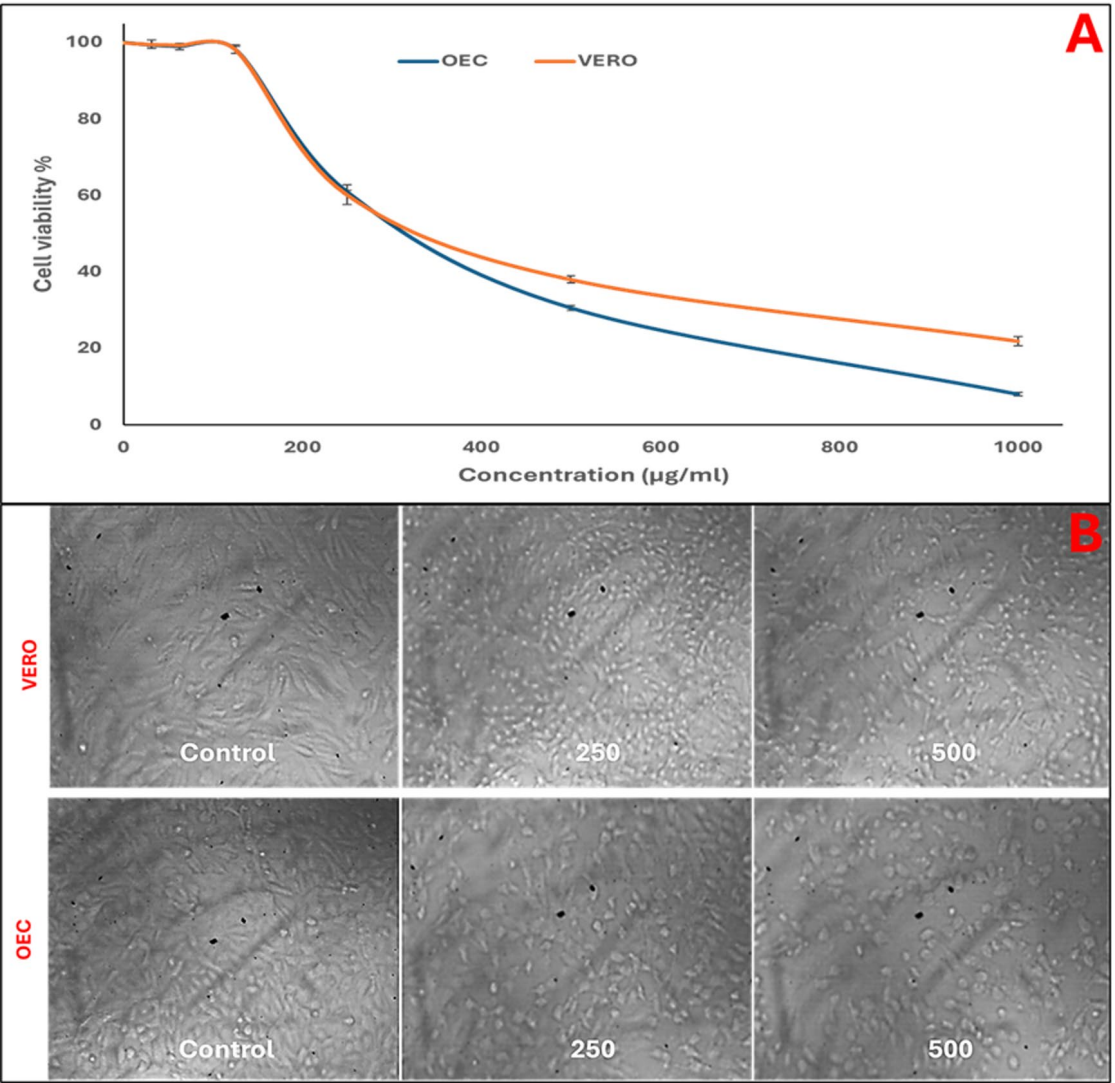
Cytotoxicity assessment of CoO-NPs on a normal cell line: (**A**) MTT assay and (**B**) morphological observations under an inverted microscope.

The cytotoxicity of CoO-NPs was evaluated against two normal cell lines, African green monkey kidney (VERO) and oral epithelial cells (OEC). The results revealed a dose-dependent decrease in cell viability, with IC₅₀ values of 303.52 µg/mL for VERO cells and 253.19 µg/mL for OEC cells (Fig. 10A). Morphological observations under the inverted microscope supported these findings: control cells of both lines exhibited a healthy, confluent monolayer with normal morphology. Treatment with 250 µg/mL CoO-NPs led to a slight reduction in cell density and minor morphological alterations, indicating some loss of viability. At 500 µg/mL, more pronounced effects were observed, including decreased cell density and altered cell shape, reflecting a clear cytotoxic response at this concentration (Fig. 10B).

The MTT assay results indicate that OEC cells are slightly more sensitive to CoO-NPs than VERO cells. Nevertheless, both IC₅₀ values exceed the 100 µg/mL threshold commonly used to classify low-toxicity materials, suggesting that CoO-NPs exhibit a relatively safe profile under the tested conditions^69,70^. These observations are consistent with previous studies reporting low cytotoxicity of cobalt-based nanoparticles toward normal cells. For example, cobalt metal nanoparticles showed minimal toxicity against human embryonic kidney (HEK) cells, implying that they may be less harmful to normal cells compared to cancerous cells^71^.

CoO-NPs, like other metal oxide nanoparticles, can induce concentration-dependent reductions in cell viability through mechanisms including oxidative stress, reactive oxygen species (ROS) generation, and membrane damage. Previous studies on cobalt oxide systems have demonstrated that higher nanoparticle exposure correlates with decreased mitochondrial activity and overall cell viability^72^.

Moreover, nanoparticle characteristics strongly influence biological responses. Smaller nanoparticles generally possess a higher surface-to-volume ratio, enhancing interactions with cellular membranes and facilitating uptake, often resulting in greater cytotoxicity compared to larger particles. Shape and morphology also modulate interactions, with certain geometries promoting easier internalization or increased membrane perturbation. Surface charge (zeta potential) further affects electrostatic interactions with negatively charged cell membranes: positively charged nanoparticles typically exhibit higher cellular affinity and greater cytotoxic potential relative to neutral or negatively charged counterparts^73,74^.

## Conclusion

This study demonstrates the successful green synthesis of **CoO-NPs** using *Salvia officinalis* extract, yielding crystalline nanoparticles of 10–50 nm. The CoO-NPs exhibited strong antibacterial activity against ESBL-producing *E. coli* and *K. pneumoniae*, with MIC values of 0.312–0.625 mg/mL, and notably enhanced the efficacy of several conventional antibiotics, including rifampicin, meropenem, and gentamicin, highlighting their potential as antibiotic adjuvants. The nanoparticles also showed moderate antioxidant activity in DPPH and ABTS assays and acceptable cytotoxicity against normal cell lines, with IC₅₀ values indicating a favorable therapeutic window. These results suggest that *S. officinalis*-mediated CoO-NPs are a promising therapeutic agent combining antimicrobial efficacy with biocompatibility. Future studies should investigate their mechanisms of antibacterial action, broader activity against multidrug-resistant pathogens, in vivo safety and efficacy, and optimization of green synthesis for scalable production with consistent properties.

## Data Availability

All data generated or analyzed during the study, and raw data can be obtained from the corresponding author upon reasonable request.

## Abbreviations

AK: Amikacin
AM: Amoxicillin
AMC: Amoxicillin/Clavulanic acid
ATCC: American Type Culture Collection
CAZ: Ceftazidime
CIP: Ciprofloxacin
CN: Gentamicin
CoO-NPs: Cobalt oxide nanoparticles
CRO: Ceftriaxone
CTX: Cefotaxime
DPPH: 2,2-diphenyl-1-picrylhydrazyl
ESBL: Extended-spectrum β-lactamase
F: Nitrofurantoin
FEP: Cefepime
FTIR: Fourier Transform Infrared Spectroscopy
G+ ve: Gram-positive
G-ve: Gram-negative
HR-TEM: High-Resolution Transmission Electron Microscopy
IC_50_: Inhibitory concentration 50
LB: Luria Bertani
LEV: Levofloxacin
MEM: Meropenem
MHB: Muller-Hinton broth
MIC: Minimum inhibitory concentration
MIFE: Madhuca indica flower extract
OD: Optical density
RA: Rifampin
RM-ANOVA: Repeated measures ANOVA
RT: Retention times
SAM: Ampicillin-Sulbactam
SPR: Surface plasmon resonance
SXT: Sulfonamide-trimethoprim
TPZ: Piperacillin-Tazobactam
XPS: X-ray Photoelectron Spectra
